# Local Nursing Home Prescribing Patterns and Psychoactive Prescribing in Assisted Living

**DOI:** 10.1093/geroni/igaa057.2485

**Published:** 2020-12-16

**Authors:** Kali Thomas, Christopher Wretman, Philip Sloane, Anna Beeber, Paula Carder, Lindsay Schwartz, Sheryl Zimmerman, Johanna Silbersack

**Affiliations:** 1 Brown University, Providence, Rhode Island, United States; 2 University of North Carolina at Chapel Hill, Chapel Hill, North Carolina, United States; 3 Portland State University, Portland, Oregon, United States; 4 American Health Care Association/ National Center for Assisted Living, Pittsboro, North Carolina, United States; 5 University of North Carolina at Chapel Hill

## Abstract

Because prescribing practices in long-term care settings may reflect regional influences, we examined how potentially inappropriate antipsychotic and antianxiety medication prescribing in assisted living (AL) compared to prescribing in nursing homes (NHs) based on their proximity, using generalized linear models adjusting for facility characteristics and state fixed effects. Data were derived from a seven state sample of AL communities and data for the same seven states drawn from publicly available data reported on the Nursing Home Compare website. In adjusted analyses, AL rates of antipsychotic use were not associated with the rates in the nearest or farthest NHs. However, AL communities that were affiliated with a NH had lower rates of potentially inappropriate antipsychotic use (b=−0.27[95%CI=−0.50,−0.04]). In a separate model, antianxiety medication prescribing rates in AL were significantly associated with neighboring NHs’ rates of prescribing (b=2.65[95%CI=1.00,4.29]). Findings suggest efforts to change prescribing in NHs may influence prescribing in AL.

